# Utilizing perspectives from HIV-infected women, male partners and healthcare providers to design family planning SMS in Kenya: a qualitative study

**DOI:** 10.1186/s12913-019-4708-7

**Published:** 2019-11-21

**Authors:** Karren Lewis, Elizabeth K. Harrington, Daniel Matemo, Alison L. Drake, Keshet Ronen, Gabrielle O’Malley, John Kinuthia, Grace John-Stewart, Jennifer A. Unger

**Affiliations:** 1 Department of Global Health, University of Washington, Seattle, USA; 2 Department of Obstetrics & Gynaecology, University of Washington, Harborview Medical Center, 325 Ninth Ave., Box 359909, Seattle, WA 98104 USA; 3 Department of Research and Programs, Kenyatta National Hospital, Nairobi, Kenya

**Keywords:** mHealth, Family planning, HIV, SMS

## Abstract

**Background:**

Short message service (SMS) presents an opportunity to expand the reach of care and improve reproductive health outcomes. SMS could increase family planning (FP) use through education, support and demand generation. The purpose of this analysis is to determine the perspectives of potential FP users to inform design of SMS.

**Methods:**

We conducted focus group discussions (FGD) with HIV-infected women and in-depth interviews (IDI) with male partners and health care workers (HCW) at urban and rural clinics in Kenya to design SMS content for a randomized controlled trial.

**Results:**

Women and men indicated SMS could be used as a tool to discuss FP with their partners, and help decrease misconceptions about FP. Women stated SMS could make them more comfortable discussing sensitive topics with HCWs compared to in-person discussions. However, some women expressed concerns about FP SMS particularly if they used FP covertly or feared partner disapproval of FP use. These findings were common among women who had not disclosed their status. Providers viewed SMS as an important tool for tracking patients and clinical triage in conjunction with routine clinical visits.

**Conclusion:**

Our findings suggest that SMS has the potential to facilitate FP education, counselling, and interaction with HCWs around FP.

## Background

Providing quality family planning (FP) counselling and service delivery remains a challenge to achieving optimal FP access in Sub-Saharan Africa. This is especially important among HIV-infected women of reproductive age who account for the largest proportion of HIV-infected individuals in Kenya [1]. Integration of FP into HIV services has been successful in improving access to and uptake of FP among HIV-infected individuals [2], and offers an opportunity to involve partners [3, 4]. However, despite efforts, unmet need for FP remains high; in Kenya, approximately 20% of HIV-infected women of reproductive age want to stop or delay childbearing but are not using any method of contraception [5, 6].

Previous work has shown that HIV-infected women have many of the same concerns about FP as uninfected women including partners’ disapproval [7], fertility desires [7] and concerns about side effects [7]. However, they face specific challenges, such as need for dual contraceptive use, prevention of mother to child transmission (PMTCT) [8], and interactions with antiretroviral therapy (ART) [8, 9]. Research has highlighted the complex contraceptive decision-making of couples affected by HIV [4, 7]. Thus, it is necessary to design novel approaches to FP care delivery for HIV-infected women.

Mobile health (mHealth) interventions present one approach to providing FP counselling and supporting the FP needs of HIV-infected women. mHealth solutions in which patients receive SMS messages from healthcare workers, have been shown to be effective for supplementing clinical care and improving health outcomes [10] and could be beneficial for FP. While mHealth has been widely accepted for appointment reminders and education among HIV-infected populations, [11] integration of FP-related messaging is largely understudied [12]. Studies focusing on FP health promotion or education [13] have demonstrated acceptability and feasibility but have not shown impact on FP use [14]. We aimed to design a culturally appropriate SMS project with input from the end-user community.

We conducted a formative study to inform FP-related SMS content for a randomized controlled trial (RCT) of tailored 1-way SMS versus 2-way SMS dialogue to improve maternal adherence to antiretroviral therapy (ART) (Option B+) and retention in care in Kenya [15]. We hypothesize that integrating FP messaging for HIV-infected peripartum women into an SMS strategy for Option B+ would improve integration of clinical care, provide efficiencies in messaging and present a more holistic approach to healthcare for this population, leading to improved outcomes. Our objectives were to investigate perceptions of SMS to support FP use and to design FP-focused SMS messages for the RCT.

## Methods

For the formative phase of an ongoing RCT in Kenya (Mobile WACh-X) [15] participants were recruited from three public sector clinics with an HIV prevalence of 15–19%. This selection of clinics included both rural and peri-urban settings. Human subject approvals were obtained from the University of Washington and Kenyatta National Hospital, and all study participants provided written informed consent. All interview guides were developed for this study and are available as Additional file 1.

**Fig. 1:**
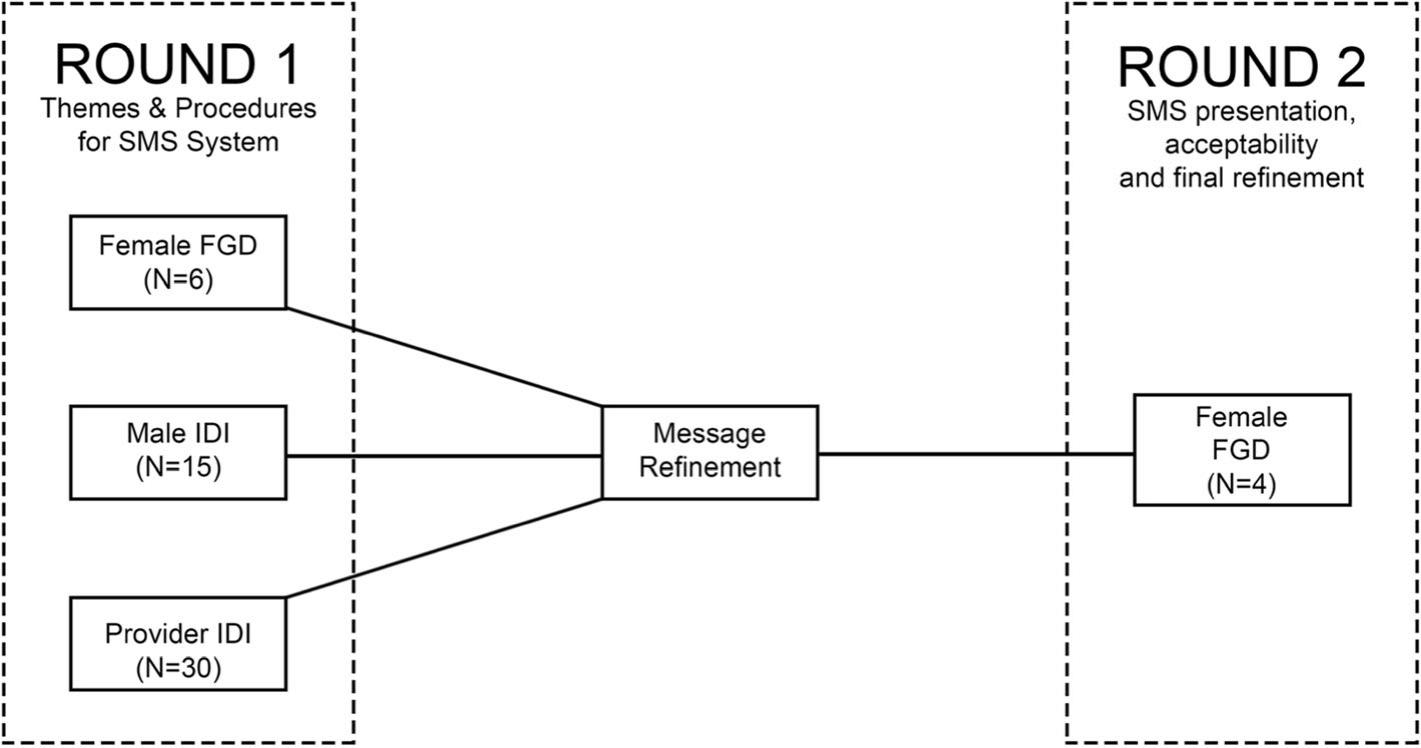
Schematic of formative phase interviews and message refinement

Women were recruited by study staff from perinatal support groups and ANC clinics. Women were purposively sampled to include both pregnant and postpartum women with varying ART experience (Table 1). Female participants completed a sociodemographic survey and were scheduled for a focus group discussion (FGD) at a later date. Two rounds of FGDs, with each focus group containing between 7 and 10 participants, (*N* = 87 total participants) took place in each location with groups conducted in English, Swahili and Luo (Fig. 1). Six FGDs took place in the first round and 4 FGDs in the second round. In the first round an experienced facilitator used a semi-structured guide developed for this study (sumplementary files) to address topics such as challenges in care utilization, medication adherence, and FP. She also explored themes for additional messaging concerns, such as security and message sharing. The facilitator distributed example SMS, some containing FP messaging, developed for this intervention, to elicit feedback and facilitate discussion. SMS messages were refined following round 1, and in round 2, the facilitator presented new SMS to explore comprehension, acceptability and further refine content.

**Table 1:** HIV experience among female FGD participants

	HIV Experience		
Site	ART outside of pregnancy	ART for PMTCT only	No prior ART experience
Rural site 1	14	5	5
A rural site	24	0	1
Mathare	22	14	2
Total	60	19	8

Male participants were recruited through female FGD participants and by study staff who recruited men with HIV-infected female partners from each study site (5 men per site, *N* = 15). Men completed a sociodemographic survey and interviews were conducted by the same facilitator addressed: SMS comprehension and acceptability, involvement of male partners in SMS programs primarily targeting women, potential use and acceptability of SMS to discuss sensitive topics such as HIV and FP, and general concerns about SMS communication.

Health care providers, including nursing officers, maternal child health (MCH) nurses, clinical officers, pharmacists and peer counsellors, were recruited to participate and given a survey prior to an in-depth interview (IDI). Each site enrolled 10 providers (*N* = 30). IDI topics discussed included: current provider challenges to HIV and MCH care; and perceived potential for SMS to improve clinic attendance, retention in care, HIV education, and communication of sensitive topics such as FP.

Interviews were transcribed from audio recordings and translated into English when necessary. Transcripts and survey data were analysed in Dedoose (Los Angeles, CA) [16]. Two investigators (KL and EH) coded each set of transcripts. After independently reading transcripts, they developed an initial list of codes for each transcript set. Preliminary codebooks, along with an open coding process to incorporate emerging thematic codes, were used to code five transcripts in each set; EH and KL then compared codes and developed revised codebooks for each set of transcripts. The final codebook was reviewed with a senior qualitative expert (GO). All transcripts were then coded or re-coded, and EH and KL met to compare code application and consistency, and reach consensus. Textual data was then grouped by code and transcript set to allow for comparing and contrasting themes across FGDs and IDIs to emerge. Overarching themes were synthesized into memos or thematic summaries. Memos were then reviewed by members of the Mobile WACh X team to refine emerging concepts.

## Results

Female FGD participants (*n* = 87) had a median age 26 years (interquartile range [IQR]: 23–31.5 years), were predominantly in monogamous partnerships (66%), and most had children (93%). The majority (74%) completed primary education, and most (91%) had experience with ART/PMTCT. Median age of men (*n* = 15) was 37 years (IQR 33–43 years) and most (93%) lived with their current partner. The majority (73%) of men received at least some primary education and reported as HIV-infected (80%); all HIV-infected men had prior or current ART experience. Health care providers (*n* = 30) had a median of 6 years in clinical practice (IQR: 4–15 years) (Table 2).

**Table 2:** Participant Characteristics

	Women (*n* = 87)	Men (*n* = 15)	Providers (*n* = 30)
Site			
Rural site 1	24	5	10
Rural site 2	26	5	10
Mathare	37	5	10
Demographics			
Age median, years, (IQR)	26 (23–32)	37 (33–43)	36 (31–43)
In relationship = n (%)	75 (86)	15 (100)	28 (93)
Married n (%)	70 (80)		27 (90)
Education n (%)			
Less than primary	23 (26)	3 (20)	
At least primary	43 (49)	8 (53)	
Secondary or above	20 (23)	4 (27)	
Pregnant	30 (34)		

Both women and men generally favored SMS as a strategy to improve knowledge, dispel myths and empower women to initiate FP. A married woman, aged 20–24, from a rural site said, ‘[If] you send SMS I can know that family planning is good for my health: especially now that I know my status I should plan my family.’ Since SMS messages could be shared with male partners, many women felt FP-related SMS could be a tool to educate partners about FP options, and help dispel common FP misperceptions for both partners. A married 35–39 year old woman from a rural site said, ‘[If] you want to use family planning, then you should educate yourselves with your husband so that none of you gets surprised’. Women said it would be important for both partners to receive SMS messages on women’s health because they could help clarify recommendations from clinic visits for FP decision-making.

I think it [SMS] can be sent because it is good even the spouse would understand it [IUCD], it is easy to put, it takes many years, it easy to remove and at any time it is good if he sees that it can be removed easily (married woman (age 30-34), rural site).

Women also noted that SMS offered a level of anonymity that could help overcome barriers to discussing sensitive topics like FP:

Sending messages can be easier to tell my story. Some things are difficult to just face someone and tell them, like you can find it hard to tell the doctor that your husband has refused to use condoms … Even if you have problems with your breast or private parts you will have problems telling the doctor of opposite sex, but through SMS you can tell him … and you will get help (married woman (age 20-24) , urban site).

Male participants echoed the sentiments about the potential for SMS to educate and dispel FP myths, and described how messaging could help change men’s opinions. Several men described the need for repeated delivery of health information to help them overcome widespread misperceptions.

[My] wife has been encouraging me about family planning … but I have not understood … SMS which encourages family planning should be sent to me also to read … I will eventually understand it and agree to it (HIV-infected male (age 25-29), rural site).

Women who reported disclosing their HIV status to partners were more interested in detailed SMS that could be both educational and supportive to them and their partners. Several described the act of disclosure as a gateway to increased communication about health topics. A married woman, aged 25–29, from the urban site said, ‘I had not disclosed to my husband, so I was just hiding … One day … he saw the drugs. He took it and read, so I just told him [and] I started to cry. But he understood and we went together and got tested.’ The importance of openness and communication within couples was repeatedly mentioned in relation to discussions about FP messaging. Some women felt that HIV disclosure and discussion of FP were mutually reinforcing:

[If] my husband [knows] my status then he will be the first person to advise me to go for family planning. (married woman (age 20-24, rural site).

Although most participants appreciated the potential for SMS to support FP, partner HIV status disclosure figured prominently in relation to women’s opinions about SMS for FP. Women who had not disclosed their HIV status were generally not comfortable receiving SMS with overt messaging about HIV or FP, and tended to express a higher level of concern about the SMS content. Women mentioned various consequences of unintended HIV disclosure, such as physical abuse, relationship dissolution, and loss of financial support. Several women described using ART without the knowledge of their male partners, and concerns about concealed FP use being discovered as a result of partners reading FP-related SMS on their phones.

Most] men have not embraced family planning, in that case if he receives the message and reacts badly then it will mean that he hasn’t been told about … family planning, since most men do not like family [planning] … if you use it secretly it might affect you (married woman, (age 25-29) urban site).

Women felt strongly about obtaining permission prior to sending FP messaging, in order to help protect privacy. A married woman, age 25–29, from a rural site said, ‘Some men have issues with family planning, and they don’t allow their wives to use it, so … you use it in secret … I think that it [FP-related SMS messages] can be sent to someone who has consented.’

Overall, men showed a strong interest in being included in FP-related SMS messaging as part of a clinic-based intervention. However, some men articulated concerns about FP messages causing problems for women in the community whose partners had misperceptions about FP including female infidelity. One man said:

There are some men who don’t want anything to do with family planning, so when you want to send SMS you have to try as hard as possible for them [women] to share ideas about family planning with their husbands so that they get to a point where the husband knows what it is all about (HIV-infected male (age 25-29), rural site).

Participants identified limitations to sending FP-related SMS, but an especially important one for providers was the inability of SMS to deliver counselling on complex topics and to deliver FP services. Both women and providers said SMS could not replace attending clinic, in-depth counselling and determining medical eligibility.

[No] we cannot use another SMS to counsel; because she has to be there in person … [The] mother has to come to the hospital [to] check the weight and blood pressure. She cannot just come and it [implant] is inserted, she has to undergo examination before it is inserted (MCH nurse (age 55-59), rural site).

Several health care providers expressed concern about confidentiality and covert FP use with use of SMS. A mentor mother, age 45–49, in the urban site said, ‘I don’t want you to mention … the kinds of family planning practice because these clients don’t want [their] partner to know what it is … they use the family planning without even telling the partner.’ They also noted that women should have to consent to receiving FP-related messages.

Providers described several ways in which SMS could improve clinic flow and clinical care in general. First, they felt it was an important way to remind women about appointments and track missed visits. Additionally, SMS were thought to act as a mechanism to triage complaints and avoid unnecessary visits to clinic.

I think the provider workload will reduce, because some of them come to the clinic specifically because they have some questions … ‘When do I start family planning?’ … You can just SMS and tell the lady, ‘don’t come this time it is not yet’...instead of wasting time to come here (nurse (age 20-24), rural site).

## Discussion

This study explores the perspectives of key stakeholders, including HIV-infected women, male partners, and health care providers, on the utility of SMS for FP education and support. Unmet need for family planning among HIV-infected women in Kenya remains high, making innovative strategies to reach this population a high priority [1]. We identified important considerations for the development of SMS programs tailored for HIV-infected women in the setting of an mHealth intervention focusing on Option B+/lifelong ART adherence.

Experience with HIV disclosure was a strong determinant of women’s desire for messaging with HIV or FP-related content. Previous studies support the potential for HIV disclosure to lead to improved communication about HIV and other health topics within sexual relationships [17]. Furthermore, HIV disclosure is postulated to have important effects on health care outcomes; specifically, non-disclosure has been associated with poorer PMTCT outcomes [18], likely due to limitations on health-seeking behaviours. In our study, women who had not disclosed their HIV status had concerns not only about HIV-related messaging, but also about receiving SMS with FP content. While HIV disclosure may open the door to couple communication about other sensitive topics, it is also possible that women in more gender-equitable relationships are more comfortable talking with partners about these topics, including HIV and FP. A qualitative study of couples in Kenya supports this idea that couple communication and trust are prerequisites for HIV disclosure [19]. As communication and power dynamics within couples influence women’s comfort with, and participation in, mHealth programs, these complex issues need to be considered in SMS development and how mHealth interventions are advertised to women.

Although SMS interventions have demonstrated benefits in HIV care outcomes [10] as well as uptake of MCH care [20], there is also potential for harm by inadvertent disclosure of personal information through SMS. Messaging interventions targeting health outcomes in low-resource settings often employ a “one size fits all” approach [10, 14, 21]. Our data suggest that programs directed towards women accessing HIV and RHFP information should include tailored options to elect the type of messages they want to receive, and provide clear communication about the content of messages, especially in the context of phone sharing. As a result of this formative work, we implemented example message demonstrations as part of the study introduction and consent process. In addition, we added a transition SMS message which informed women about and introduced the subsequent FP messaging that would follow. The introduction message read *“Over the next few weeks we will send you messages to help you make family planning decisions. Planning your family and spacing pregnancy is good for your health.”* In this way, women were aware that the next few messages would be counselling about FP.

Within heterosexual relationships, it is important to consider the role of men in FP decision making. Previous research in the region suggests that FP education in the clinical setting may not be an ideal way to shift male resistance to FP [22], and community-based interventions, which may include SMS, may be a more viable option. Our findings highlight varying perspectives among HIV-infected women regarding men’s response to FP-related messaging. Most women and men perceived benefits to sharing FP-related SMS with partners, and felt male partners should be included in SMS programs. Most participants viewed SMS as an acceptable method to engage men and prior studies demonstrate the importance of male inclusion in FP discussions for successful FP programs [3, 23]. As a result we did include messages about speaking to partners about family planning. An example of this type of message read: *“Spacing your pregnancies keeps you and your family healthy. Partners are sometimes against family planning or do not have enough information. If you feel comfortable, please share the messages with your partner. Family planning is very safe. Return to fertilty is very quick with most methods! Are you using family planning? Is your partner supportive?”.* Yet, a small number of women had concerns about potential negative effects, indicating that this approach is not universally acceptable.

In addition, SMS may help improve patient-provider relationships and increase clinic efficiency. As in other studies, interviews with healthcare workers demonstrated interest in using SMS for ART adherence support and appointment tracking [24], triaging patient complaints, and improving patient-provider communication. SMS may also be able to alleviate fears women have about talking to providers in person due to previous experiences with mistreatment [25] or concerns discussing sensitive topics. While many benefits of SMS were articulated by participants, healthcare providers and women both stressed the necessity for clinic-based counselling and inability of SMS to replace clinical services.

As program implementers increasingly recognize the promise of SMS in education and behaviour change, and mHealth programs utilizing SMS grow in number, this study’s findings support the need to elucidate community desires and concerns prior to roll-out. This formative work informed the design and content of FP messaging, and more broadly, altered our approach to the study consent process regarding HIV messaging [26]. Women in the associated RCT have more control over what types of HIV-related messages they choose to receive.

Our study has some limitations. As an exploratory qualitative study focused on HIV-infected women, our results may not be generalizable to other populations. FP-related experiences and concerns among HIV-infected women may differ from those with unknown or HIV negative status. In addition, views and experiences of women engaged in HIV care may not reflect the views from people not involved in care. Men in this study were primarily recruited via referral from female partners, which may bias the results towards male support for FP-related messaging.

## Conclusion

This study provides insight into the perspectives of HIV-infected women, men, and providers on an SMS approach to FP education, and emphasizes the need for community input during development of mHealth programs. Without appropriate formative work, programs might inadvertently send FP or HIV-related messaging that place women at risk—or contribute to conflict within couples. Our study demonstrated SMS is an acceptable approach to delivering FP education. However, community context and social acceptability of SMS topics must be identified and appropriate consent obtained, if applicable. In many settings, designing programs to include both women and men may be beneficial. SMS appears to be an acceptable bridge to improve communication between patients and providers; further research is needed to determine if SMS interventions can lead to improved FP uptake and explore impact on FP-related communication between women and their partners.

## Supplementary information

**Additional file 1.** Focus group discussion guides.

## Data Availability

The data sets used and analysed during the current study are available from the corresponding author upon request.

## Abbreviations

ART: Antiretroviral therapy
FGD: Focus group discussion
FP: Family planning
HCW: Health care worker
IDI: In-depth interview
MCH: Maternal child health
mHealth: Mobile health
PMTCT: Prevention of mother to child transmission
RCT: Randomized controlled trial
SMS: Short message service
